# MY11 exerts antitumor effects through activation of the NF-κB/PUMA signaling pathway in breast cancer

**DOI:** 10.1007/s10637-022-01272-0

**Published:** 2022-06-27

**Authors:** Qun Ye, Ziwei Jiang, Ying Xie, Yuanhong Xu, Yiyi Ye, Lei Ma, Lixia Pei

**Affiliations:** 1 Institute of Chinese Traditional Surgery, Longhua Hospital Affiliated to Shanghai University of Traditional Chinese Medicine, Shanghai, 200032 China; 2 Shanghai Key Laboratory of New Drug Design, School of Pharmacy, East China University of Science and Technology, Shanghai, 200237 China

**Keywords:** Chalcones, NF-κB, PUMA, Apoptosis, Cell cycle, Breast cancer

## Abstract

Breast cancer is the most common malignancy in women worldwide, and the discovery of new effective breast cancer therapies with lower toxicity is still needed. We screened a series of chalcone derivatives and found that MY11 ((E)-1-(2-hydroxy-4,6-dimethoxyphenyl)-3-(4-piperazinylphenyl) prop-2-en-1-one) had the strongest anti-breast cancer activity. MY11 inhibited the growth of MDA-MB-231 and MCF-7 breast cancer cells by arresting the cell cycle and promoting apoptosis, through regulation of the cell cycle and apoptosis-related proteins. PDTC (Pyrrolidinedithiocarbamate ammonium), a specific inhibitor of the NF-κB pathway, abolished the inhibitory effect of MY11 treatment. NF-κB has been shown to regulate PUMA-dependent apoptosis. Our *in vitro* studies demonstrated that MY11 promoted breast cancer cell apoptosis by activating the NF-κB/PUMA/mitochondrial apoptosis pathway (including Bcl-2, Bax, and Caspase-9). MY11 also inhibited tumor growth in an orthotopic breast cancer mouse model by inducing apoptosis through the NF-κB signaling pathway, importantly, with minimal toxicity. In addition, MY11 was found by docking analysis to bind to p65, which might enhance the stability of the p65 protein. Taken together, our findings indicate that MY11 exerts a significant anticancer effect in breast cancer and that it may be a potential candidate for the treatment of breast cancer.

**Supplementary information:**

The online version contains supplementary material available at 10.1007/s10637-022-01272-0.

## Introduction

Breast cancer is the most commonly diagnosed cancer worldwide, accounting for approximately 24.5% of new cancer cases and 15.5% of deaths in women [1, 2]. Although advances in drug treatment have reduced breast cancer mortality, drug resistance and side effects still limit therapeutic options [3]. There is still an urgent need to find novel therapeutic agents with high efficacy and few side effects for the treatment of breast cancer.

Effective elimination of cancer cells by promoting apoptosis is a strategy for tumor therapy [4]. The Bcl-2 (B-cell lymphoma-2) family is one of the gene families most highly involved in apoptosis and functions as a ‘life/death switch’ to determine whether the mitochondrial stress-induced apoptotic pathway should be activated [5, 6]. This family includes Bcl-2, Bax and the ‘‘BH3-only’’ group of proteins [7]. Bax is a proapoptotic protein that forms toxic oligomers to permeabilize the mitochondrial outer membrane and thus induce downstream apoptosis events, including the activation of caspase signaling cascades [8]. Bcl-2 exerts an antiapoptotic effect by heterodimerizing with Bax and disrupting the permeabilization pore [9, 10]. It has been shown that overexpression of PUMA (p53 upregulated modulator of apoptosis), a BH3-only protein, promotes apoptosis in various cancer cells [11–13]. When induced by apoptotic stimuli, PUMA binds to Bcl-2, releases Bax, and ultimately activates caspase signaling cascades [14].

The NF-κB signaling pathway regulates important physiological processes, such as cell growth, differentiation and apoptosis [15, 16]. The NF-κB family includes the p50/p105, p52/p100, p65 (or RelA), c-Rel and RelB subunits, which dimerize to form functional protein complexes [17]. After stimulation, NF-κB rapidly separates from IκB and translocates into the nucleus to regulate the transcription of target genes [18]. NF-κB has been reported to either induce or inhibit apoptosis under different conditions. There is evidence that NF-κB directly regulates PUMA and induces PUMA-dependent apoptosis in vitro and in vivo [19, 20].

The bud of *Cleistocalyx operculatus* is a traditional Chinese herb that has a long history of use in tonic drinks [21]. DMC (2',4'-dihydroxy-6'-methoxy-3',5'-dimethylchalcone) is a natural chalcone isolated from this bud that has shown pharmacological effects, such as anti-inflammatory, antihyperglycemic and anticancer activities [22]. Previously, starting with the scaffold of DMC, Yin et al. designed a series of chalcone derivatives and found that the compound MY3 had the greatest ability to reverse DOX resistance in MCF-7 cells [23]. However, whether these derivatives have direct antitumor activities remains unknown.

In this study, we screened a series of DMC-engineered chalcone derivatives and found that the compound MY11 ((E)-1-(2-hydroxy-4,6-dimethoxyphenyl)-3-(4-piperazinylphenyl) prop-2-en-1-one) showed the greatest inhibitory potency against breast cancer cells in vitro. Hence, we explored the underlying anticancer mechanism of MY11. Further studies showed that MY11 not only induced cell cycle arrest and apoptosis in breast cancer cells but also activated the NF-κB/PUMA signaling pathway. More importantly, we demonstrated that MY11 also inhibited the growth of breast cancer in an orthotopic breast cancer mouse model by activating the NF-κB/PUMA signaling pathway. Our results indicate that MY11 could be a promising candidate drug for breast cancer treatment.

## Materials and methods

### Reagents

MY11 (purity 95%) was synthesized and provided by pharmaceutical chemistry laboratory, East China University of Science and Technology, China [23]. RPMI 1640 and DMEM was purchased from Hyclone (Logan, UT, USA). MEM, Penicillin/Streptomycin and Fetal Bovine Serum (FBS) were obtained from Gibco (Invitrogen Corporation, New York, USA). Antibodies for Cyclin D1 (A19038), PUMA (A3752), Bcl-2 (A19693), Bax (A19684), NF-κB p65 (A19653), Phospho-NF-κB p65-S536 (AP0124), β-Actin (AC004) and secondary antibodies were purchased from ABclonal (Wuhan, China). Caspase-9 (#9502) and p21 (#2947) antibodies were purchased from Cell Signaling Technology (Danvers, MA, USA). FITC Annexin V Apoptosis Detection Kit were purchased from BD Biosciences Company. SYTOX Red dead cell stain was purchased from Invitrogen. SYBR green master mix was purchased from Vazyme. Solutol HS-15 was obtained from MedChemExpress. Hematoxylin and Eosin Staining Kit were purchased from Beyotime.

### Cell culture

Human breast cancer cell lines MDA-MB-231, MCF-7 and mouse breast cancer cell line 4T1 were purchased from the Cell Bank of the Chinese Academy of Sciences (Shanghai, China). MDA-MB-231 cells were cultured in DMEM medium supplemented with 10% FBS and 100 U/mL Penicillin/Streptomycin. MCF-7 cells were maintained in MEM supplemented with 10% FBS, 1% sodium pyruvate, 0.01 mg/ml human recombinant insulin and 100 U/ml Penicillin/Streptomycin. 4T1 cells were cultured in RPMI 1640 medium supplemented with 10% FBS and 100 U/mL Penicillin/Streptomycin. All the cells were maintained at 37 °C in a humidified incubator containing 5% CO_2_.

### MTT assay

Cells were seeded into 96-well plates at a density of 4 × 10^3^ cells each well and then treated with different concentrations of MY11 for 12 h. Subsequently, 20 µL MTT solution (5 mg/mL) was added into each well and the plates were incubated at 37 ℃. After 4 h, the medium was discarded, and the dark blue crystal on the bottom was dissolved completely with 150 µL DMSO. The absorbance was measured at the wavelength of 490 nm using Synergy H1 Hybird Reader (Bio Tek Instruments Inc., Winooski, Vermont, USA). The IC_50_ values of MY11 in MDA-MB-231, MCF-7 and 4T1 cells were calculated using the SPSS 21.0 software (Abbott Laboratories, Chicago, USA).

### Colony formation assay

MCF-7 and MDA-MB-231 cells were seeded into 6-well plates at a density of 1 × 10^3^ cells each well. Cells were treated with different doses of MY11 for 7 h after attachment and incubated for another 24 h. The resultant cell colonies were stained with 1% crystal violet for 10 min and the numbers of colonies were counted for analysis.

### Cell cycle assay

After treated with different concentrations of MY11 for 12 h, cells were fixed with 75% ethanol overnight, and stained with Sytox Red staining buffer (PBS containing 5 μM Sytox Red, 0.2 mg/mL RNase, and 0.1% TritonX-100) for 15 min at room temperature. Cell cycle was detected by the D × FLEX Flow Cytometer.

### Cell apoptosis assay

After treated with different concentrations of MY11 for 12 h, cells were harvested and washed twice by PBS. Fluorescence of MY11 was detected to overlap with PI and 7AAD. Therefore, Annexin V-FITC and Sytox Red (excitation and emission same as APC) were used to stain cells for 15 min in the dark at room temperature and the apoptosis rates of cells were detected by the D × FLEX Flow Cytometer (Beckman Coulter, Fullerton, CA, USA).

### Western blot analysis

Protein was extracted from cells using RIPA lysis buffer (Beyotime, China) containing protease inhibitor cocktail and phosphatase inhibitor cocktail (APExBIO, Houston, USA) and its concentration was detected with BCA assay (Beyotime, China). 20 μg of total protein from each sample or PageRuler Prestained Protein Ladder (Thermo Fisher Scientific, Waltham, MA, USA) was diluted in loading buffer (Beyotime, China) for immunoblot. Proteins were separated with SDS-PAGE and transferred to a nitrocellulose blotting membrane. Membranes were blocked in 5% BSA for 1.5 h, and incubated with primary antibodies overnight. Then membranes were washed by PBST and continued to be incubated with HRP-conjugated secondary antibodies for 1 h. The protein bands were visualized by ECL Enhanced Kit (ABclonal, China) and detected by Bio-Rad ChemiDoc™ Touch Imaging System. Antibodies against p21, Cyclin D1, PUMA, Bcl-2, Bax, Caspase-9, NF-κB p65 and Phospho-NF-κB p65-S536 were used at a dilution of 1: 1,000. β-Actin and secondary antibodies were used at a dilution of 1: 8,000.

### Real-time PCR

The cells were harvested after treatment and total RNA was extracted with RNAiso Plus (Takara Bio Inc., Japan). Total RNA was used for cDNA synthesis using HiScript II Q RT SuperMix for qPCR (Vazyme Biotech Co.,Ltd, Nanjing, China) according to the manufacturer’s protocol. Real-time PCR was performed in triplicate using SYBR green master mix on a QuantStudio 3 System (Vazyme Biotech Co.,Ltd, Nanjing, China). The samples with low yield of RNA were pre-determined and excluded. Quantitative analysis was performed using 2-ΔΔCt method for quantification of the relative mRNA expression.

Real-time PCR specific primers were designed and validated by NCBI Primer designing tool (https://www.ncbi.nlm.nih.gov/tools/primer-blast/) and all primers are shown as follows. hGAPDH (forward: 5'-CTTAGCACCCCTGGCCAAG-3'; reverse: 5'-TGGTCATGAGTCCTTCCACG-3'); hCyclin D1 (forward: 5'-ATCAAGTGTGACCCGGACTG-3'; reverse: 5'-CTTGGGGTCCATGTTCTGCT-3'); hp21 (forward: 5'-TGCCGAAGTCAGTTCCTTGT-3'; reverse: 5'-GTTCTGACATGGCGCCTCC-3'); hBcl-2 (forward: 5'-CTGCACCTGACGCCCTTCACC-3'; reverse: 5'-CACATGACCCCACCGAACTCAAAGA-3'); hBax (forward: 5'-GACATTGGACTTCCTCCGGG-3'; reverse: 5'-ACAGGGACATCAGTCGCTTC-3'); mGAPDH (forward: 5'-CTTAGCCCCCCTGGCCAAG-3'; reverse: 5'-TGGTCATGAGCCCTTCCACA-3'); mCyclin D1 (forward: 5'-CAGCCCCAACAACTTCCTCT-3'; reverse: 5'-CAGGGCCTTGACCGGG-3'); mp21 (forward: 5'-TATCCAGACATTCAGAGCCACA-3'; reverse: 5'-ACTTTGCTCCTGTGCGGAA-3'); mBcl-2 (forward: 5'-CTGAGTACCTGAACCGGCAT-3'; reverse: 5'-AGTTCCACAAAGGCATCCCAG-3'); mBax (forward: 5'-AAACTGGTGCTCAAGGCCC-3'; reverse: 5'-CTTGGATCCAGACAAGCAGC-3').

### Molecular docking

Molecular docking was employed to analyze the binding potential between p65 and MY11. MY11 was constructed by ChemDraw, and optimized by Chem3D. The structure of the target protein p65 was downloaded from the protein database, the RCSB database (https://www.rcsb.org/structure/). The processing and optimization of virtual screening is executed using Glide module in Schrödinger Maestro software. Protein Preparation Wizard module is used for protein processing. The receptor was pretreated, optimized and minimized (constraint minimization using OPLS3e force field). All compounds were prepared according to the default settings of the LigPre module. When screening in Glide module, the prepared receptor is introduced to specify the appropriate location in receptor grid generation. The protoplast of the protein was selected as the centroid of the 10 Å box. OPlS3e is selected for the relay field, and SP (Standard Precision) is selected as the docking method. Finally, analyze the interaction mode of compounds and proteins, extract the docking score of compounds to speculate whether the compounds to be screened have certain activity.

### siRNA transfection

MDA-MB-231 and MCF-7 cells were cultured in antibiotics free medium in 6-well plates for 24 h before siRNA transfection. The cells were transfected with 10 µL 20 µM siRNA using 5 µL Lipofectamine 2000 reagent for 4 h according to the protocol of Lipofectamine. Subsequently, transfection reagents were discarded and antibiotics-free medium was added into each well. The transfected cells were cultivated for another 24 h and then subjected to following experiments. The siRNA sequences used were as following: si-p65-1 (sense: 5'-GGACAUAUGAGACCUUCAAdTdT-3'; antisense: 5'-UUGAAGGUCUCAUAUGUCCdTdT-3'); si-p65-2 (sense: 5'-GAUGAAGACUUCUCCUCCAdTdT-3'; antisense: 5'-UGGAGGAGAAGUCUUCAUCdTdT-3'); si-p65-3 (sense: 5'-CCUAUGUGGAGAUCAUUGAdTdT-3'; antisense: 5'-UCAAUGAUCUCCACAUAGGdTdT-3').

### Animal experiments

Six weeks old female BALB/c mice were bought from Zhejiang Vital River Laboratory Animal Technology. Mice were housed in a pathogen-free (SPF) environment at 22–23 ℃ with food and water supplied ad libitum throughout the experimental period. All experiments were performed under the ARRIVE Guideline (animal pre-clinical studies) approved by Institutional Animal Care and Use Committee (IACUC) of Longhua Hospital affiliated to Shanghai University of Traditional Chinese Medicine. After mice were anaesthetized with Isoflurane, 2 × 10^4^ 4T1 cells resuspended in 100μL of PBS and Matrigel (1:1) were injected into the right mammary fat pad of each mouse. One week later, mice were divided into five groups: normal group, vehicle control group (ultrapure water mixed with Solutol HS-15 in a 9:1 ratio), MY11 20 mg/kg group, MY11 30 mg/kg group, MY11 40 mg/kg group. MY11 were diluted in ultrapure water containing Solutol HS-15 (9:1). Treatment for all groups were administered by intraperitoneal injection every day. The tumor size and body weight were measured every 3 days. Tumor volumes were calculated by the formula: Length × Diameter^2^)/2. After three weeks of treatment, mice were sacrificed, and all tumors and organs were harvested, measured and subjected to following experiments. The expression of Cyclin D1, p21, PUMA, Bcl-2, Bax, Caspase-9, NF-κB p65 and Phospho-NF-κB p65-S536 in the tumors were detected by Western blot.

Serum ALT and AST levels were measured using the ALT Kit and AST Kit (Nanjing Jiancheng Bioengineering Institute, China), respectively. Briefly, 10 μL serum was added into ALT or AST reagent. The optical density was measured at the wavelength of 505 nm using Synergy H1 Hybird Reader (Bio Tek Instruments Inc., Winooski, Vermont, USA). Each sample was measured in triplicate.

### Hematoxylin and eosin (H&E) staining and Immunohistochemistry (IHC)

Tissue morphology was analyzed by H&E staining. 4% PFA was used to fix the tumor for 24 h. Sections (5 μm) of paraffin-embedded tumor tissue specimens were prepared on glass slides. The sections were subsequent stained with Hematoxylin and eosin. For immunohistochemical of Ki67 in tumors, five micrometers of formalin-fixed, paraffin-embedded tissue sections were mounted on glass slides. The sections were performed deparaffinization, rehydration, antigen retrieval and endogenous peroxidase inactivation. After blocking, the sections were stained with anti-mouse Ki67 antibody (A16919, ABclonal) overnight, then incubated with the HRP-labeled Goat anti-Rabbit IgG (H + L) antibody (A0208, ABclonal). Ki67 antibody were used at a dilution of 1: 200. After DAB staining and hematoxylin counterstaining, sections were dehydrated with graded alcohol, hyalinized in xylene, and finally sealed with neutral gum. Histologic analyses were observed and photoed by Olympus Microscope. (Tokyo, Japan), the positive cells were stained brown.

### Statistical analysis

Statistical analyses were carried out using the SPSS 21.0 software and GraphPad Prism 7 software. One-way or two-way analysis of variance (ANOVA) was used to analyze the significance between the groups. All values were mean ± standard error of the mean and were representative of three independent experiments, and *P* < 0.05 was considered statistically significant.

## Results

### MY11 inhibits the growth of human breast cancer cells

**Fig. 1:**
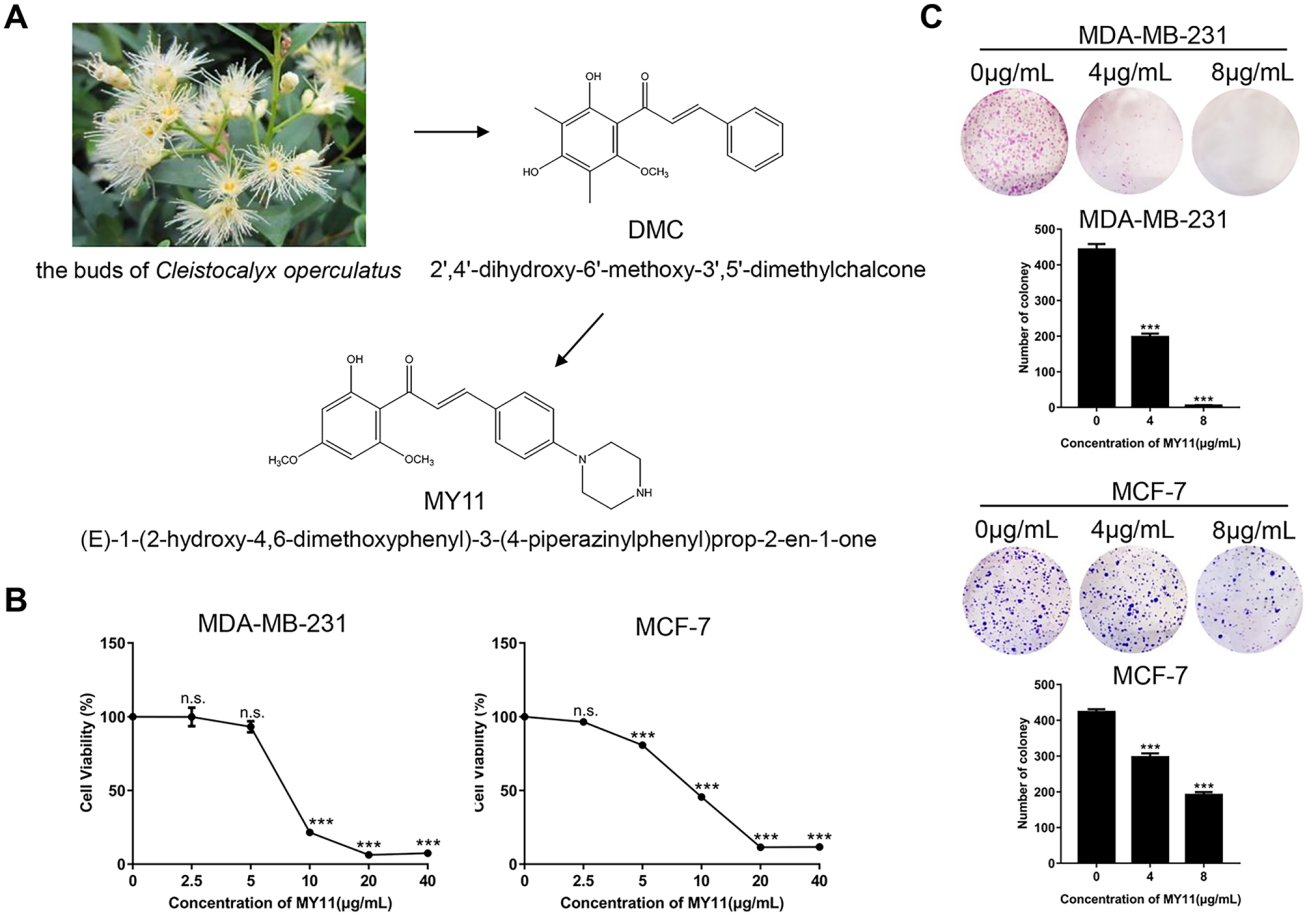
MY11 inhibits the growth of human breast cancer cells. **A** Chemical structure of DMC and MY11. **B** MTT assay for cell viability of MDA-MB-231 and MCF-7 cells treated with different concentrations of MY11 for 12 h. **C** Growth inhibition effects of MY11 on MDA-MB-231 and MCF-7 cells were measured by colony formation assay. Bar graphs showed the quantitative results of the colony formation assay (below). Data are mean ± standard error of the mean and are representative of three independent experiments. *p < 0.05, **p < 0.01, ***p < 0.001 vs. control

To identify potential molecules with anti-breast cancer activity, we first used an MTT assay to screen a series of DMC-derived compounds (synthesized by the pharmaceutical chemistry laboratory at the East China University of Science and Technology, China. Structures shown in Supplemental Fig. 2A, C). As shown in Supplemental Fig. 2B, D, MY3, MY11 and MC7 inhibited the proliferation of MDA-MB-231 breast cancer cells. We chose MY11 for further research (Fig. 1A) and confirmed its growth inhibition effect in MCF-7 cells. As shown in Fig. 1B, the half maximal inhibitory concentration (IC_50_) of MY11 was 8 μg/mL (22 μM) in MDA-MB-231 cells and 9 μg/mL (24 μM) in MCF-7 cells when they were treated for 12 h. In addition, MY11 significantly decreased the number of colonies formed by both cell lines when applied at 4 and 8 μg/mL (Fig. 1C). Taken together, these results indicated that MY11 inhibited the growth of human breast cancer cells.

### MY11 induces cell cycle arrest in breast cancer cells

**Fig. 2:**
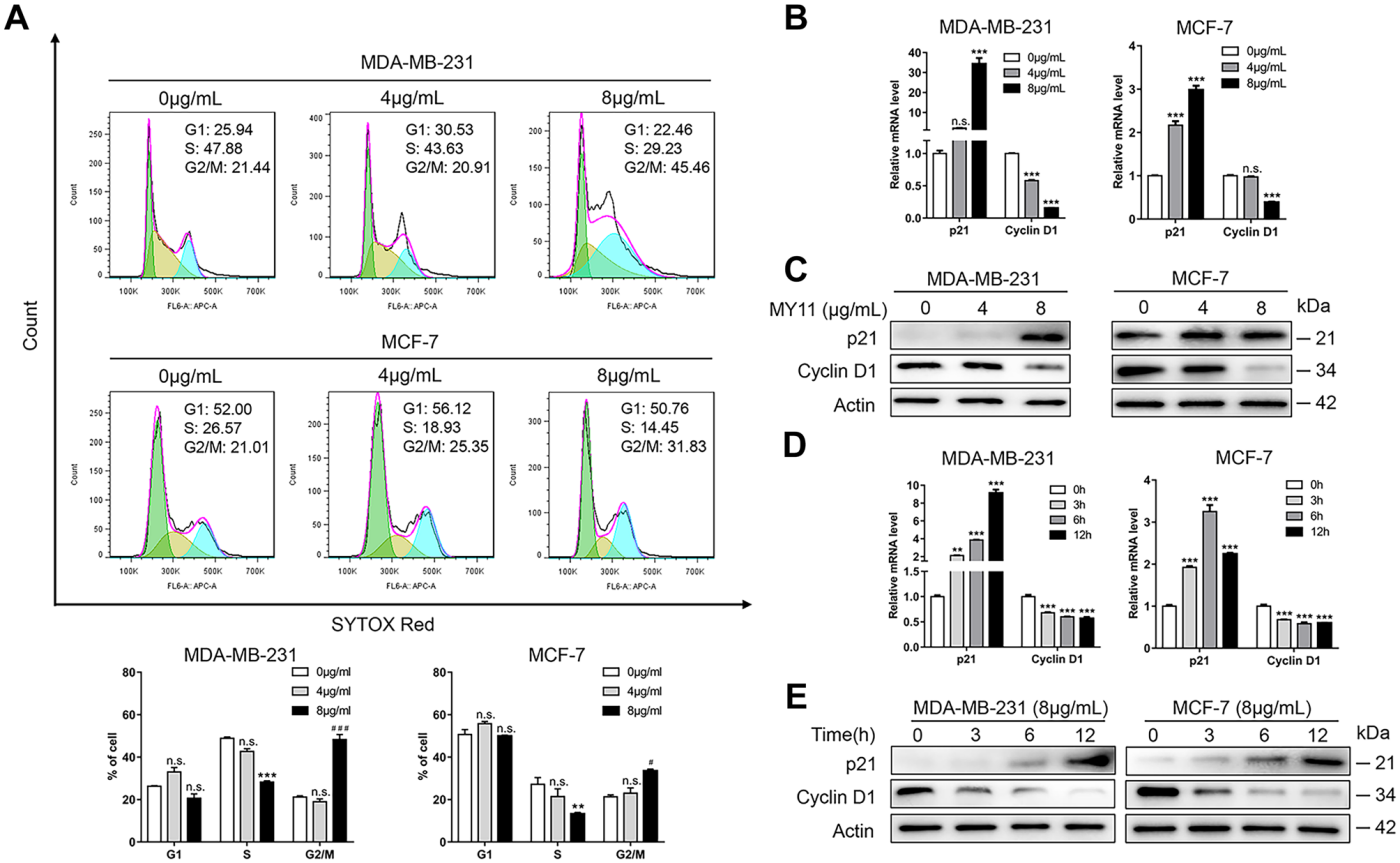
MY11 induces cell cycle arrest in breast cancer cells. **A** Flow cytometry analysis of the cell cycle distribution after treatment with the indicated concentration of MY11 for 12 h. The percentages of cells in the G1, S and G2/M phases were presented statistically (below). **B** Real-time PCR and **C** western blot for the levels of cell cycle-related mRNA and proteins in cells treated with the indicated concentration of MY11 for 12 h, respectively. **D** Real-time PCR and **E** western blot for the levels of cell cycle-related mRNA and proteins in cells treated with MY11 (8 μg/mL) for the indicated time, respectively. Data are mean ± standard error of the mean and are representative of three independent experiments. *p < 0.05, **p < 0.01, ***p < 0.001 vs. control

To investigate the mechanism of MY11-mediated cell growth inhibition, the cell cycle distribution was assessed by flow cytometry. As shown in Fig. 2A, MY11 induced G2/M arrest in both MDA-MB-231 and MCF-7 cells when applied at 8 μg/mL for 12 h. Moreover, MY11 upregulated the expression of p21 and downregulated the level of Cyclin D1 in both MDA-MB-231 and MCF-7 cells in a dose-dependent (Fig. 2B, C) and time-dependent (Fig. 2D, E) manner, as demonstrated by both real-time PCR and Western blot analysis. These data suggested that MY11 inhibited breast cancer cell proliferation by inducing cell cycle arrest.

### MY11 induces apoptotic cell death in breast cancer cells

**Fig. 3:**
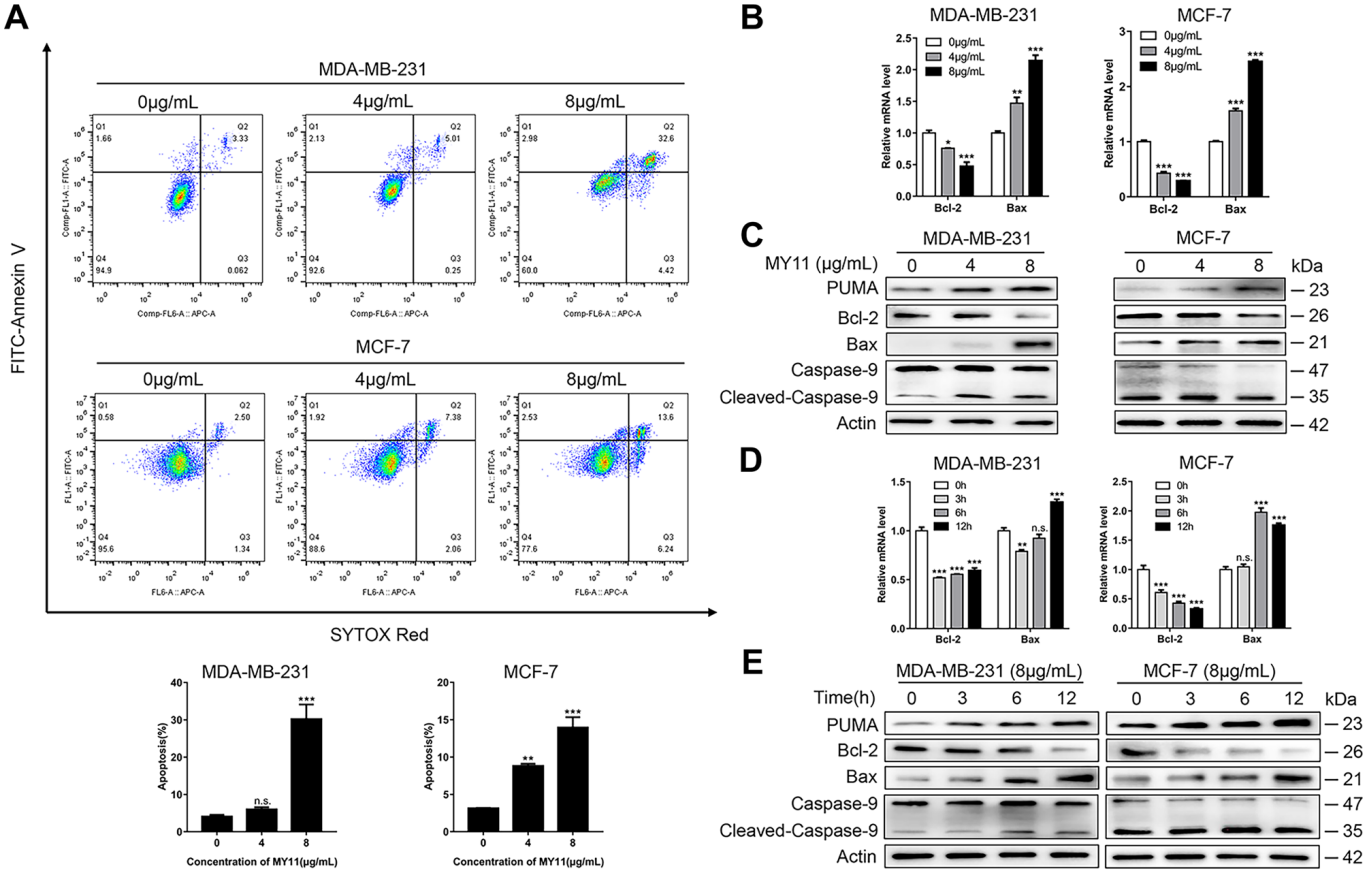
MY11 treatment induces cell apoptosis in breast cancer cells. **A** Annexin V-FITC/SYTOX Red double-staining of MDA-MB-231 and MCF-7 cells treated with the indicated concentration of MY11 for 12 h. The Annexin V-FITC/SYTOX Red double-staining was quantified and plotted below. **B** Changes in apoptosis-related mRNA and **C** proteins in cells treated with the indicated concentration of MY11 for 12 h by real-time-PCR and western blot, respectively. **D** Changes in apoptosis-related mRNA and **E** proteins in cells treated with MY11 (8 μg/mL) for the indicated time by real-time PCR and western blot, respectively. Data are mean ± standard error of the mean and are representative of three independent experiments. *p < 0.05, **p < 0.01, ***p < 0.001 vs. control

To evaluate whether the growth-inhibitory effects of MY11 are also caused by induction of apoptosis in breast cancer cells, cells stained with Annexin V and a nuclear stain were subjected to flow cytometric analysis. As shown in Fig. 3A, compared with that in the corresponding control groups, the apoptosis rate increased from 4.99% to 35.58% in MDA-MB-231 cells and from 3.08% to 16.13% in MCF-7 cells after treatment with 8 μg/mL MY11 for 12 h.

To further confirm the molecular mechanisms underlying this process, the expression of apoptosis-related proteins in MY11-treated MDA-MB-231 and MCF-7 cells was examined by Western blot analysis (Fig. 3C, E). MY11 upregulated the proapoptotic Bax and downregulated the antiapoptotic Bcl-2 in a dose-dependent (Fig. 3C) and time-dependent (Fig. 3E) manner. The downstream event of apoptosis was increased, as indicated by cleavage of the Caspase-9 protein (Fig. 3C, E). MY11 also increased the level of the BH3-only protein PUMA, which exhibits a proapoptotic effect upstream of Bcl-2 and Bax (Fig. 3C, E). The changes in Bcl-2 and Bax mRNA levels measured by real-time PCR were consistent with the Western blot data (Fig. 3B, D). Collectively, these data suggested that MY11 triggered the activation of the caspase-dependent apoptotic cascade and induced apoptosis in human breast cancer cells.

### MY11 activates the NF-κB/PUMA signaling pathway in breast cancer cells

**Fig. 4:**
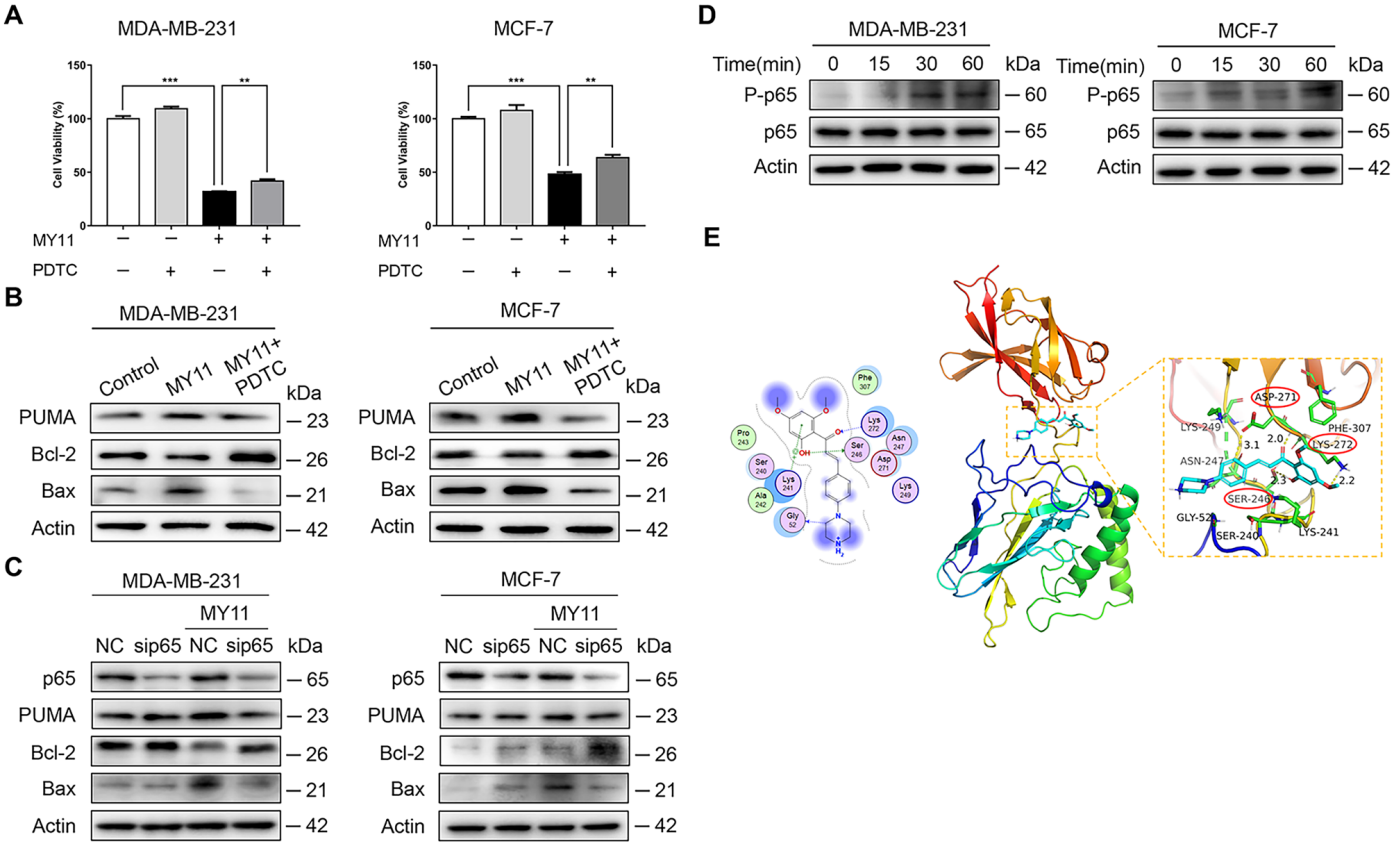
Effect of MY11 on the NF-κB signaling pathway. **A** MDA-MB-231 and MCF-7 cells were treated with or without MY11 (8 μg/mL) for 12 h in the absence or presence of PDTC (24 μM), after which the cell viability and **B** expression levels of PUMA, Bcl-2 and Bax were examined by MTT assay and Western blot, respectively. **C** MDA-MB-231 and MCF-7 cells were transfected with p65 siRNA before treated with MY11 (8 μg/mL) for 12 h. The expression of p65, PUMA, Bcl-2 and Bax was detected by Western blot. **D** MDA-MB-231 and MCF-7 cells were treated with or without MY11 (8 μg/mL) for the indicated times. NF-κB signaling-related protein levels were measured by western blot. **E** The molecular docking results of MY11 and p65 are analyzed by PyMol, and the results are graphed. Data are mean ± standard error of the mean and are representative of three independent experiments. *p < 0.05, **p < 0.01, ***p < 0.001 vs. control

To further explore the anticancer mechanism of MY11, we tested the effects of several inhibitors of well-known pathways important in cancer (Supplemental Fig. 3). Among all inhibitors, only PDTC (an inhibitor of the NF-κB signaling pathway) abrogated the growth inhibition induced by MY11 (Fig. 4A). PUMA has been reported to be directly regulated by NF-κB. Consistent with this report, we showed that PDTC reversed the MY11-induced upregulation of PUMA, as well as the changes in the downstream effectors Bcl-2 and Bax (Fig. 4B). In addition, knockdown of p65 by siRNA exhibited the same effect as PDTC treatment (Fig. 4C). To further examine the effect of MY11 on the NF-κB signaling pathway in breast cancer cells, we detected phosphorylated p65 using Western blot analysis and showed that p65 phosphorylation was effectively activated by MY11 treatment in a time-dependent manner (Fig. 4D).

To better characterize how MY11 regulates NF-κB, molecular docking was performed to analyze the affinity of MY11 for p65. The results showed that MY11 bound to p65 with high affinity, with a binding energy of -6.73 kcal/mol. According to the binding mode, the active amino acid residues of the p65 protein that bound to MY11 included ASP-271, LYS-272, SER-246, LYS-249, ASN-247, etc. MY11 formed a strong hydrogen bond interaction with amino acids in the protein active site (ASP-271, LYS-272, SER-246), with a short hydrogen bond distance (average 2.4 Å) and strong binding ability, promoting the formation of stable complexes between MY11 and the p65 protein. In addition, MY11 had a high matching degree with the protein pocket, and each region could form a strong interaction with the groove of the pocket (Fig. 4E), where the protein structure is susceptible to alterations. Therefore, this binding effectively increases the stability of this region, thereby increasing the stability of the p65 protein. Collectively, these results demonstrated that MY11 bound to p65 and activated the NF-κB/PUMA signaling pathway to regulate apoptosis-related proteins, thus inducing apoptosis in breast cancer cells.

### MY11 suppresses the growth of breast cancer cells in vivo

**Fig. 5:**
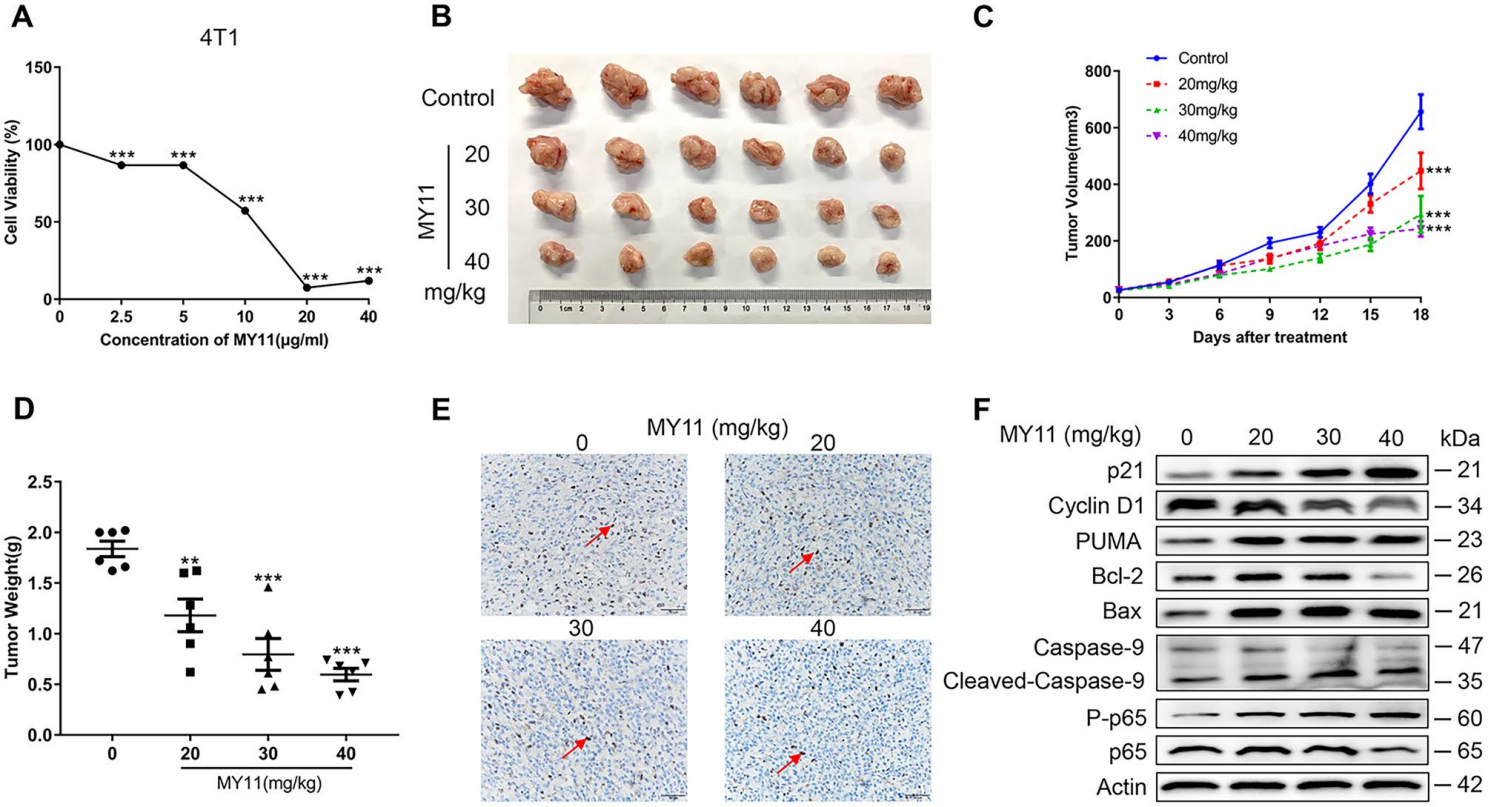
MY11 suppresses the growth of breast cancer cell in vivo. **A** MTT assay for cell viability of 4T1 cells treated with different concentrations of MY11 for 12 h. **B** Image of the tumors. **C** Average tumor volumes were measured every 3 days. **D** Average tumor weight at the end of the indicated treatment. **E** Immunohistochemical staining analysis revealed that MY11 treatment resulted in decreased proliferation (× 400 magnification). The red arrows indicate ki67 positive cells. **F** Effect of MY11 on proteins associated with NF-κB signaling, cell cycle and apoptosis in tumor tissues were detected by western blot. Data are shown as mean (± SEM) (n = 6). *p < 0.05, **p < 0.01, ***p < 0.001 vs. control (Model)

To investigate the in vivo effect of MY11 on breast cancer, we established an orthotropic breast cancer mouse model by injecting mouse breast cancer 4T1 cells into the mammary fat pads of BALB/c female mice. We first confirmed the antiproliferative effect of MY11 in 4T1 cells. As shown in Fig. 5A, the IC_50_ of MY11 in 4T1 cells was 10 μg/mL (27 μM). Moreover, MY11 upregulated the expression of p21 and Bax and downregulated the expression of Cyclin D1 and Bcl-2 in 4T1 cells (Supplemental Fig. 4A−D). Remarkably, in the 4T1 tumor-bearing mouse model, tumor growth was attenuated by MY11 treatment compared with that in vehicle-treated mice (control group) (Fig. 5B−C). The percent reductions in tumor weight relative to that in the control group were 35.8%, 56.7%, 67.6% for 20, 30, and 40 mg/kg MY11, respectively (Fig. 5D). As shown in Fig. 5E, proliferating cells in the tumor indicated by the percentage of ki67 staining were decreased by MY11 in a dose-dependent manner. Furthermore, the changes in apoptosis-related proteins and cell cycle-associated proteins, including PUMA, Bcl-2, Bax, Caspase-9, p21, and Cyclin D1, in the tumor tissues were consistent with the in vitro results. Phosphorylation of the p65 protein was upregulated by MY11, suggesting that the NF-κB signaling pathway was also activated by MY11 in vivo (Fig. 5F).

**Fig. 6:**
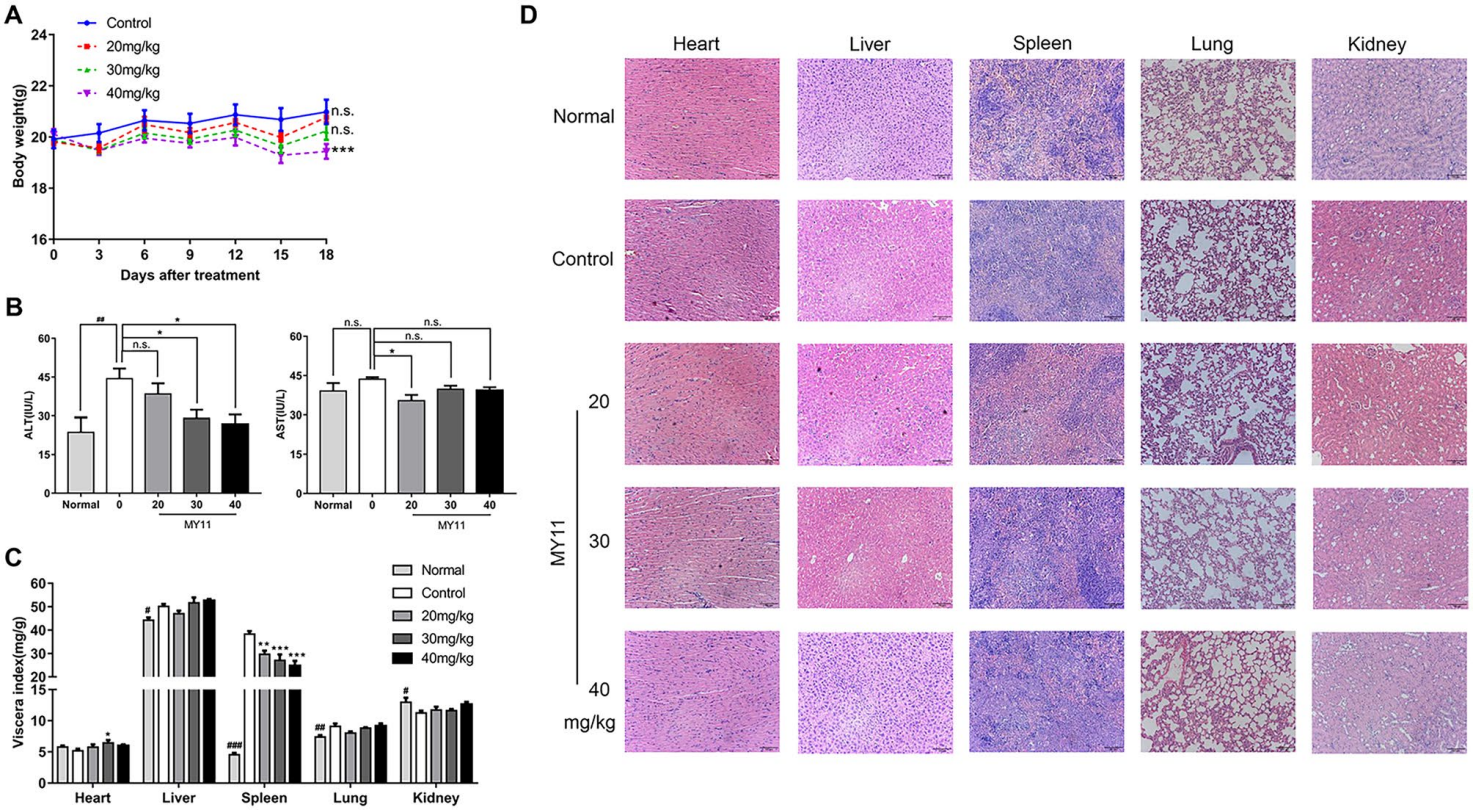
MY11 has low toxicity in vivo. **A** The body weights of the mice during the experimental period. **B** Serum ALT and AST levels in MY11-treated mice compared with the vehicle-treated mice and the normal mice. **C** Viscera index of the main organs isolated from the treated, control, and normal mice to evaluate the toxicity of MY11. **D** Hematoxylin and eosin (H&E) staining of heart, liver, spleen, lungs and kidneys collected from the mice of the treatment, the control and the normal groups (× 200 magnification). Data are shown as mean (± SEM) (n = 6). *p < 0.05, **p < 0.01, ***p < 0.001 vs. control (Model)

**Fig. 7:**
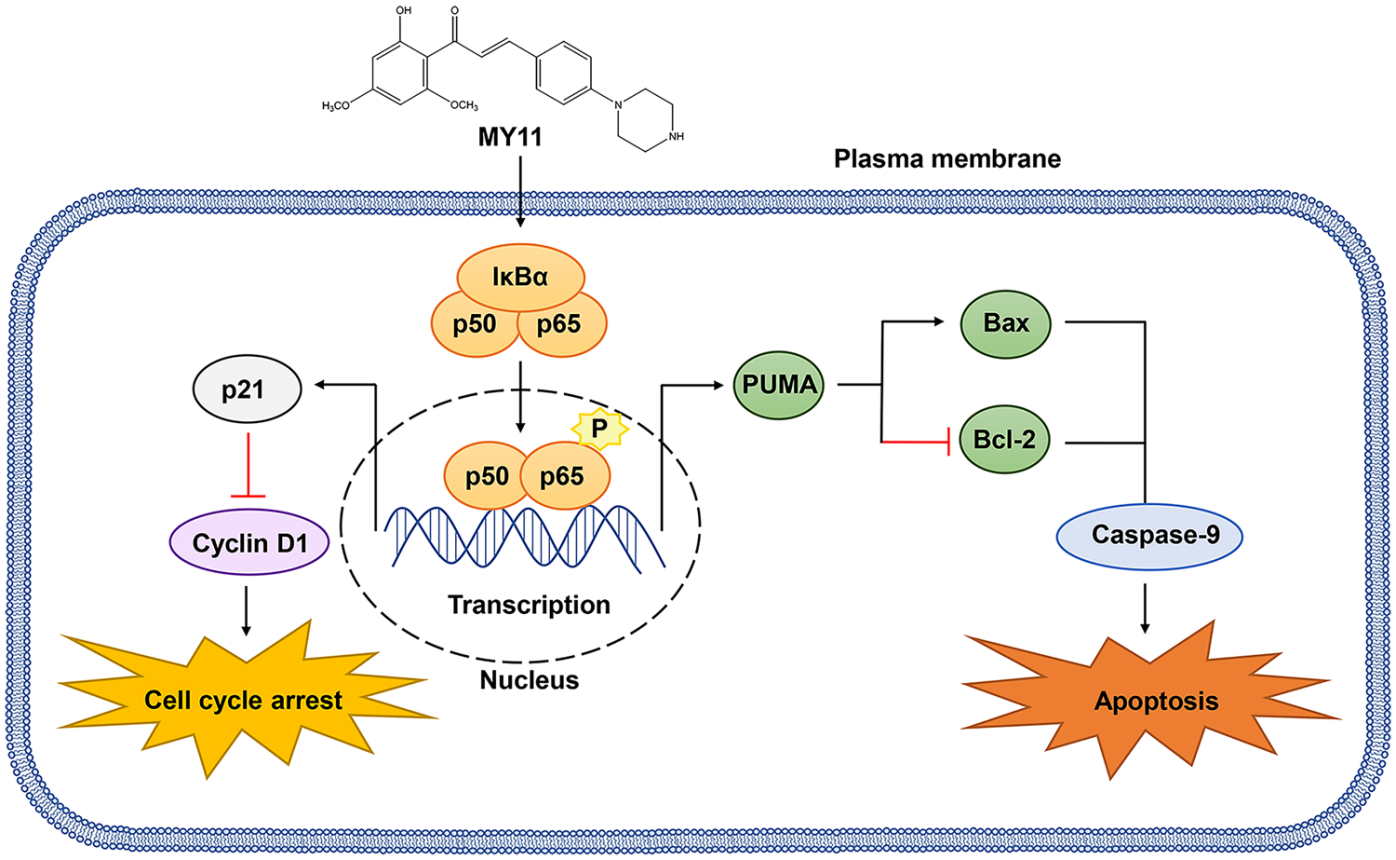
Schematic diagram summarizing the mechanisms of action of MY11 in cancer cells

In addition, we assessed the toxicity of MY11 in vivo. As shown in Fig. 6A, there were no significant differences in body weight between the treatment and control groups, except for a 7.4% reduction in the 40 mg/kg MY11 group. Serum ALT and AST levels higher than the normal reference value indicate liver damage. As shown in Fig. 6B, the serum ALT level was increased in tumor-bearing mice (control group) compared with healthy mice (normal group) and decreased upon MY11 treatment in a dose-dependent manner. There were slight or no differences in the serum AST level between the normal, control and treatment groups. In terms of visceral indices, compared with the normal group, the control group showed differences in the liver, spleen, lungs and kidneys but not in the heart. Only the spleen showed a decreasing dose-dependent trend in each dose group compared with the control group (Fig. 6C). Histological examination of vital organs, including the heart, liver, spleen, lungs, and kidneys, revealed no obvious changes in morphology (Fig. 6D). Collectively, these results indicate that MY11 is a potential anticancer agent with strong efficacy and safety (see Fig. 7).

### Discussion

Chalcones are an abundant source of antitumor derivatives with marked efficacy and low toxicity. Several promising chalcone compounds have been isolated and developed from natural products for cancer treatment. For example, Wang et al. proved that butein is an aromatase inhibitor and a potential natural alternative for the chemoprevention or therapy of breast cancer [24]. Moreover, it was reported that DMC (2',4'-dihydroxy-6'-methoxy-3',5'-dimethylchalcone) can be used for the treatment of cancer, and its IC_50_ was 270 μM in MCF-7 cells [25]. In this study, we found that the IC_50_ of the DMC-engineered chalcone derivative MY11 was 24 μM in MCF-7 cells. After structural optimization, this new chalcone derivative was much more effective in treating breast cancer. We found that MY11 induced apoptosis and cell cycle arrest in breast cancer cells and thus inhibited cell growth both in vitro and in vivo in an orthotropic breast cancer mouse model.

Cell division is mainly regulated by G1/S, S and G2/M checkpoints. DNA damage promotes G2/M checkpoint activation, which can induce cell cycle arrest while the damage is repaired. If DNA damage cannot be repaired, apoptosis eventually results [26]. Elevated levels of p21, a marker of DNA damage, delay entry into the S and M phases [27]. It is widely recognized that p21 arrests the cell cycle at the G1/S and G2/M transitions by inhibiting CDK4/CDK6/Cyclin D and CDK2/Cyclin E, respectively [28]. As shown in Fig. 2, MY11 apparently arrested MDA-MB-231 and MCF-7 cells in G2/M phase. Mechanistically, the levels of cell cycle-related proteins were significantly increased (e.g., p21) and decreased (e.g., the p21 downstream target Cyclin D1) by MY11 treatment. Our data showed that MY11 induced cell cycle arrest to suppress the proliferation of breast cancer cells.

Apoptosis is considered a highly regulated process of cell death [29]. It is an active process that sacrifices specific cells to bring greater benefits to the organism. Small molecule apoptosis inducers have been clinically used for eliminating morbid cells and treating cancer [30]. PUMA has a potent apoptosis induction effect and functions upstream of the apoptosis inhibitor Bcl-2 in the apoptosis pathway [31]. Our study indicated that MY11 markedly increased the apoptosis rate. More importantly, upregulation of PUMA, downregulation of its downstream target Bcl-2 and upregulation of Bax were detected in MY11-treated cells (Fig. 3). These results indicated that MY11 induced apoptotic cell death.

Antitumor drugs have different effects on NF-κB function to achieve the purpose of cancer treatment. For example, Sun et al. demonstrated that activation of NF-κB as a secondary pathway suppresses the growth of colon cancer under therapy with ipatasertib [20]. In contrast, Xiao et al. proved that LINC00467 is highly expressed in bladder cancer and promotes the progression of bladder cancer by activating the NF-κB signaling pathway [32]. Shanmugam et al. showed that thymoquinone inhibits NF-κB to reduce CXCR4 expression and thus has therapeutic potential in breast cancer [33]. It was previously reported that NF-κB kills cancer cells by directly inducing PUMA [34]. The p65 component of NF-κB mediates PUMA induction through a κB site in the PUMA promoter [35]. PUMA transduces death signals to mitochondria and induces mitochondrial dysfunction and caspase activation through members of the Bcl-2 family [36]. In our study, the inhibitory effect of PDTC on MY11-mediated cell death suggested that MY11 induces apoptosis through activation of the NF-κB signaling pathway. By using Western blot analysis, we demonstrated that mechanistically, PDTC reversed the changes in the expression levels of PUMA and its downstream targets Bcl-2 and Bax induced by MY11. Moreover, silencing p65 also reduced PUMA and Bax levels and increased Bcl-2 levels. Further research showed that MY11 activated the phosphorylation of p65 in a time-dependent manner. The molecular docking results also demonstrated that MY11 had a high affinity for the p65 protein (Fig. 4). Our data showed that MY11 exerted antitumor effects through activation of the NF-κB/PUMA signaling pathway.

Several studies have shown that NF-κB also induces cell cycle arrest [37]. Seitz et al. reported that NF-κB induced p21 and that this CKI (Cyclin-dependent kinase inhibitor) alone was sufficient to induce epithelial cell growth arrest [38]. In G2/M phase, all NF-κB subunits bind to the CCND1 (encoding Cyclin D1) promoter, and this binding is associated with downregulation of expression. Therefore, we concluded that MY11 induced cell cycle arrest to suppress the growth of breast cancer cells via the NF-κB signaling pathway.

Our results showed that MY11 exerted an obvious inhibitory effect on the cell proliferation and tumor growth in breast cancer in a dose-dependent manner in vitro and in vivo (Figs. 1 and 5). In animal experiments, we confirmed that the changes in the expression levels of PUMA, Bax, Bcl-2, p21, Cyclin D1 and P-p65 in tumor tissues were consistent with those observed in vitro. More importantly, MY11 treatment had little effect on the body weight, visceral indices and the morphology of vital organs, and the serum levels of ALT and AST (Fig. 6), showing its safety advantages, which is of great significance for drug development.

To summarize, herein, we found that MY11 exerted potent growth inhibitory effects in vivo and in vitro. Mechanistic analysis indicated that MY11 suppressed the expression of Bcl-2 and Cyclin D1 and induced the expression of PUMA, Bax, p21 and P-p65 in breast cancer cells in vitro and in vivo*.* Further investigation revealed that MY11 exerted its antitumor effects through activation of the NF-κB/PUMA signaling pathway. Collectively, these data provide evidence for MY11 as a potential antitumor agent for breast cancer.

## Supplementary Information

Below is the link to the electronic supplementary material.

Supplementary file1 Fig. 1 NMR spectra chromatograms of MY11. (A) ^1^H NMR (400 MHz, CDCl_3_) spectrum of MY11. (B) ^13^C NMR (150 MHz, DMSO-d_6_) spectrum of MY11 (TIF 1241 KB)

Supplementary file2 Fig. 2 Screening chalcone derivatives with antiproliferation activity of breast cancer cells. (A) Chemical structure of MY2, MY3, MY6 and MY11. (B) MTT assay for cell viability of MDA-MB-231 cells treated with different concentrations of MY2, MY3, MY6 and MY11 for 24 h, respectively. (C) Chemical structure of MC2-5, MC7. (D) MTT assay for cell viability of MDA-MB-231 cells treated with different concentrations of MC2-5 and MC7 for 24 h, respectively. Data are mean ± standard error of the mean and are representative of three independent experiments. *p< 0.05, **p< 0.01, ***p< 0.001 vs. control (TIF 932 KB)

Supplementary file3 Fig. 3 Inhibitors screen for signaling pathways that MY11 acts on. (A-D) FH535 inhibits Wnt/β-catenin signaling pathway. LY294002 and Wortmannin are PI3K inhibitors. Rapamycin is a specific mTOR inhibitor. MDA-MB-231 cells were treated with or without MY11 (8 μg/mL) for 12 h in the absence or presence of a series of signaling pathway inhibitors after which the cell viability was examined by MTT assay. Data are mean ± standard error of the mean and are representative of three independent experiments. *p< 0.05, **p< 0.01, ***p< 0.001 vs. control (TIF 1023 KB)

Supplementary file4 Fig. 4 Effect of MY11 on 4T1 cells. (A-D) Relative genes expression in MY11 treated 4T1 cells. Data are mean ± standard error of the mean and are representative of three independent experiments. *p< 0.05, **p< 0.01, ***p< 0.001 vs. control (TIF 851 KB)

## Data Availability

All data is included in this article.

## Abbreviations

NF-κB: Nuclear factor kappa-light-chain-enhancer of activated B cells
Bcl-2: B-cell lymphoma-2
IC_50_: Half maximal inhibitory concentration
DMSO: Dimethyl sulfoxide
MTT: 3-(4, 5-Dimethylthiazol-2-yl)-2, 5-diphenyl tetrazolium bromide
PDTC: Pyrrolidinedithiocarbamate ammonium
H&E: Hematoxylin and eosin staining
IHC: Immunohistochemistry
ALT: Alanine aminotransferase
AST: Aspartate aminotransferase
CKI: Cyclin-dependent kinase inhibitor
