# Supplementary figures and images for: MY11 exerts antitumor effects through activation of the NF-κB/PUMA signaling pathway in breast cancer
Source: Invest New Drugs. 2022 Jun 27;40(5):922–33. doi: 10.1007/s10637-022-01272-0 (PMC9395444; doi:10.1007/s10637-022-01272-0)

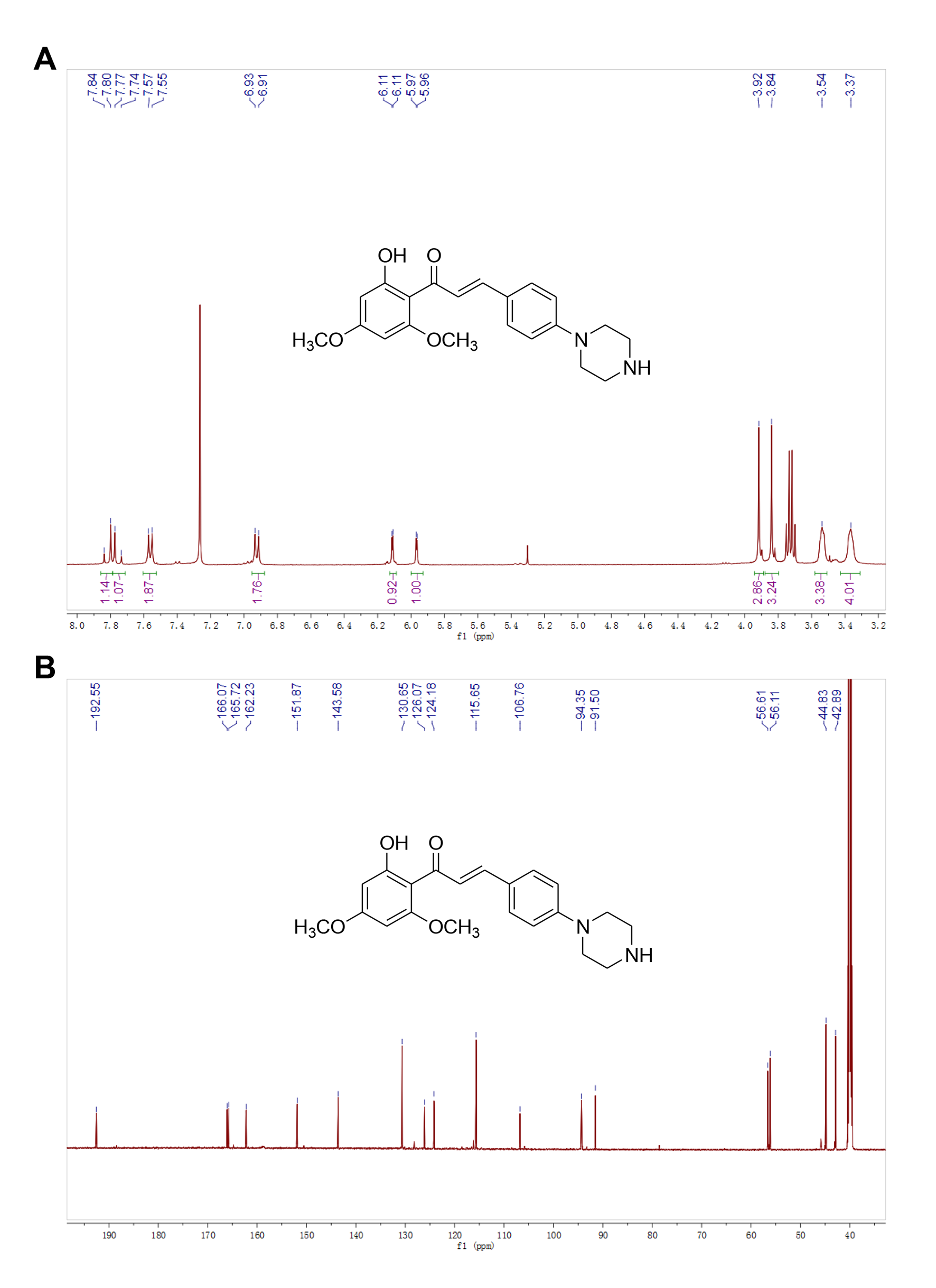

Supplement: Supplementary file 1 — Supplementary file1 Fig. 1 NMR spectra chromatograms of MY11. (A) 1H NMR (400 MHz, CDCl3) spectrum of MY11. (B) 13C NMR (150 MHz, DMSO-d6) spectrum of MY11 (TIF 1241 KB) [file 10637_2022_1272_MOESM1_ESM.tif]

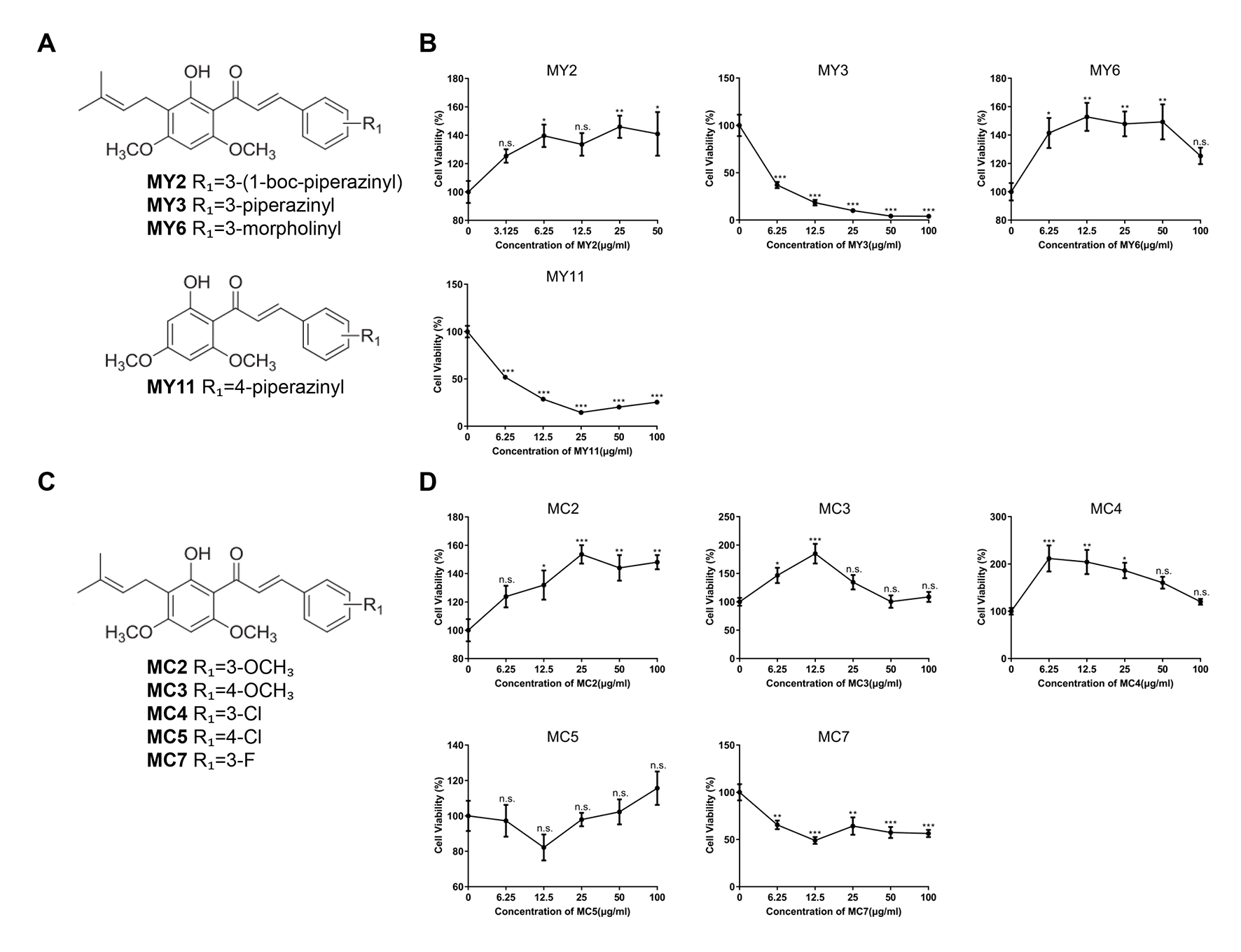

Supplement: Supplementary file 2 — Supplementary file2 Fig. 2 Screening chalcone derivatives with antiproliferation activity of breast cancer cells. (A) Chemical structure of MY2, MY3, MY6 and MY11. (B) MTT assay for cell viability of MDA-MB-231 cells treated with different concentrations of MY2, MY3, MY6 and MY11 for 24 h, respectively. (C) Chemical structure of MC2-5, MC7. (D) MTT assay for cell viability of MDA-MB-231 cells treated with different concentrations of MC2-5 and MC7 for 24 h, respectively. Data are mean ± standard error of the mean and are representative of three independent experiments. *p< 0.05, **p< 0.01, ***p< 0.001 vs. control (TIF 932 KB) [file 10637_2022_1272_MOESM2_ESM.tif]

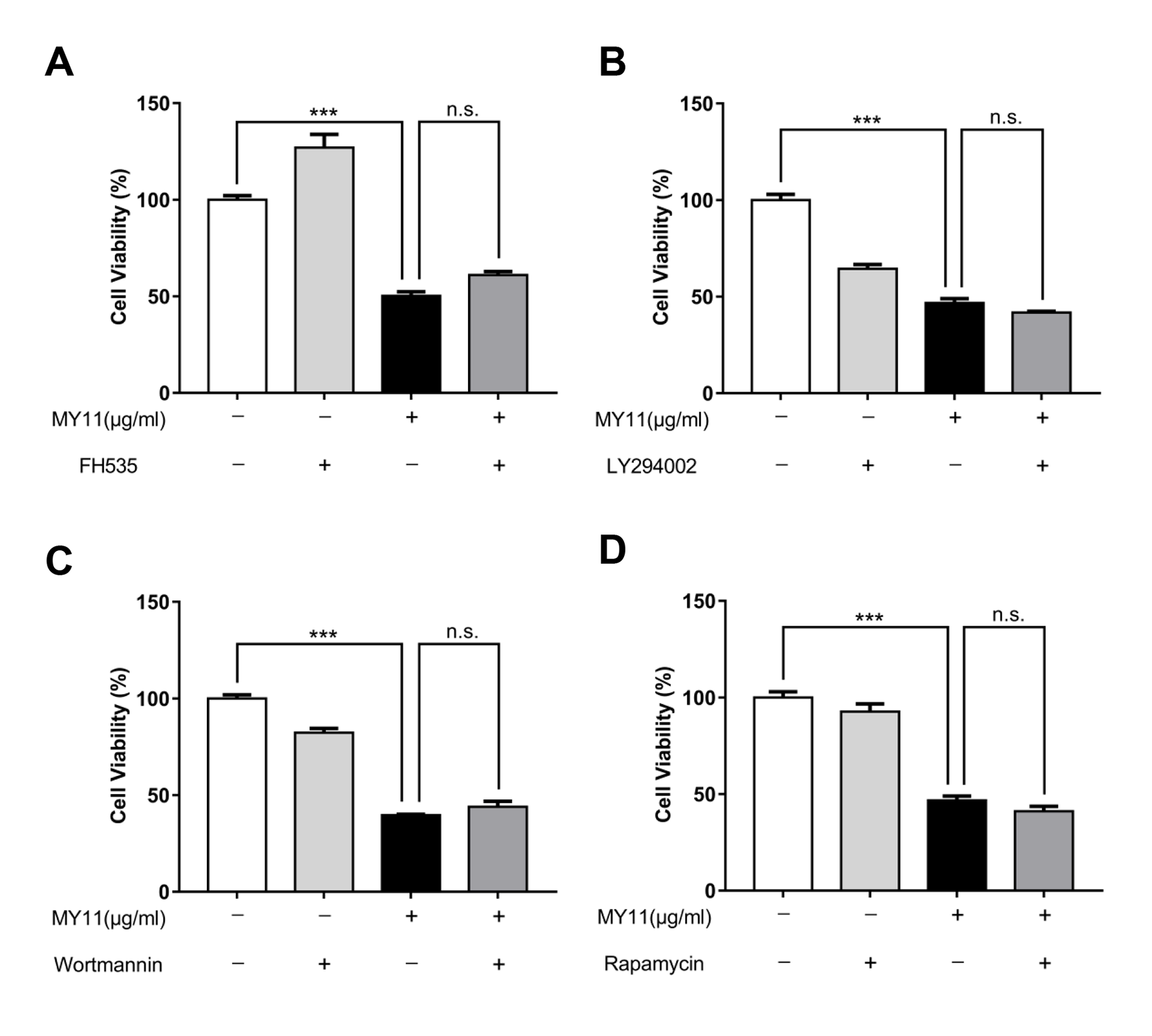

Supplement: Supplementary file 3 — Supplementary file3 Fig. 3 Inhibitors screen for signaling pathways that MY11 acts on. (A-D) FH535 inhibits Wnt/β-catenin signaling pathway. LY294002 and Wortmannin are PI3K inhibitors. Rapamycin is a specific mTOR inhibitor. MDA-MB-231 cells were treated with or without MY11 (8 μg/mL) for 12 h in the absence or presence of a series of signaling pathway inhibitors after which the cell viability was examined by MTT assay. Data are mean ± standard error of the mean and are representative of three independent experiments. *p< 0.05, **p< 0.01, ***p< 0.001 vs. control (TIF 1023 KB) [file 10637_2022_1272_MOESM3_ESM.tif]

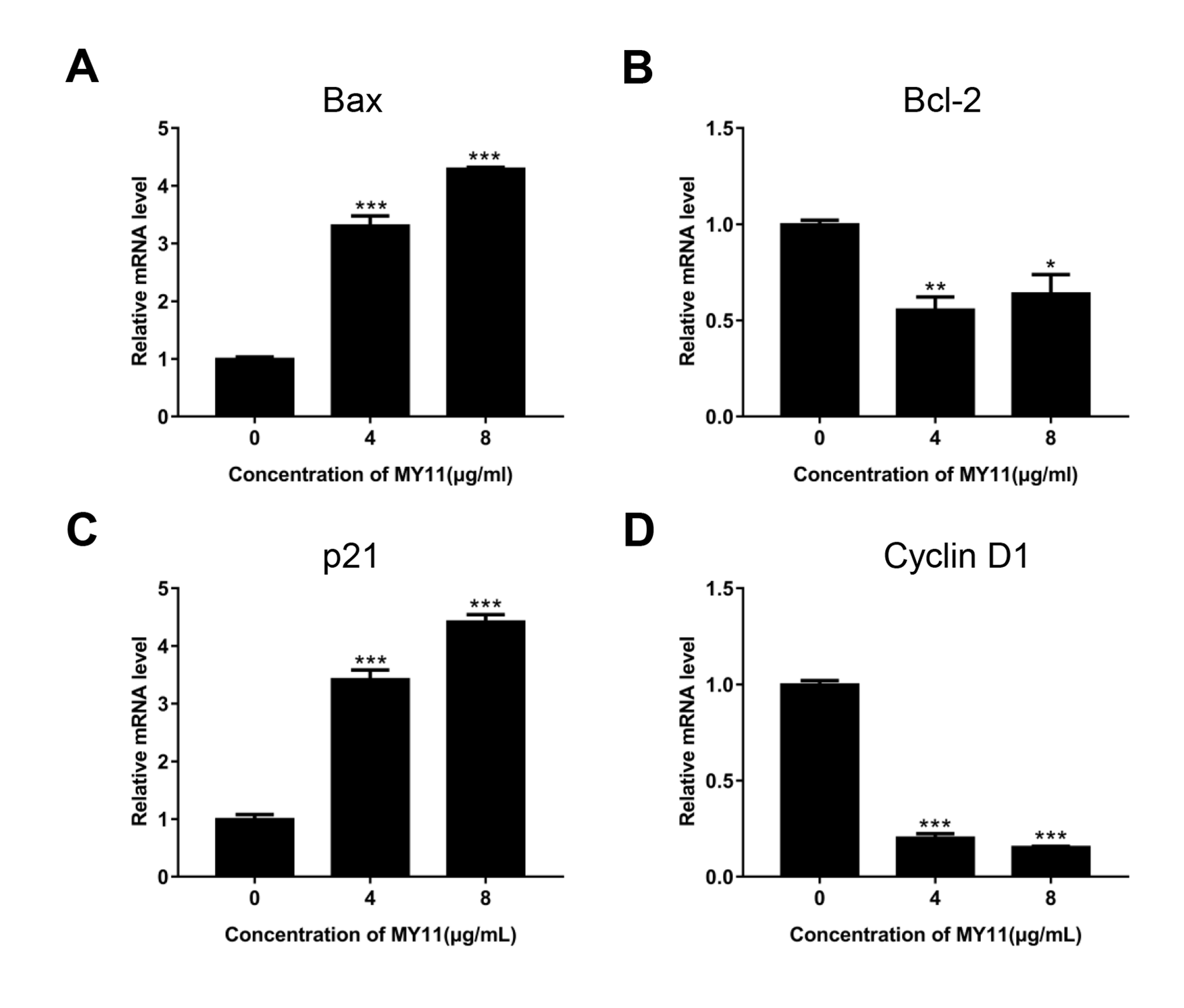

Supplement: Supplementary file 4 — Supplementary file4 Fig. 4 Effect of MY11 on 4T1 cells. (A-D) Relative genes expression in MY11 treated 4T1 cells. Data are mean ± standard error of the mean and are representative of three independent experiments. *p< 0.05, **p< 0.01, ***p< 0.001 vs. control (TIF 851 KB) [file 10637_2022_1272_MOESM4_ESM.tif]
